# Layered SiC Sheets: A Potential Catalyst for Oxygen Reduction Reaction

**DOI:** 10.1038/srep03821

**Published:** 2014-01-22

**Authors:** P. Zhang, B. B. Xiao, X. L. Hou, Y. F. Zhu, Q. Jiang

**Affiliations:** 1 Key Laboratory of Automobile Materials, Ministry of Education, and Department of Materials Science and Engineering, Jilin University, Changchun 130022, China; 2 Institute for Advanced Materials, and School of Materials Science and Engineering, Jiangsu University, Zhenjiang 212013, China

## Abstract

The large-scale practical application of fuel cells cannot come true if the high-priced Pt-based electrocatalysts for oxygen reduction reaction (ORR) cannot be replaced by other efficient, low-cost, and stable electrodes. Here, based on density functional theory (DFT), we exploited the potentials of layered SiC sheets as a novel catalyst for ORR. From our DFT results, it can be predicted that layered SiC sheets exhibit excellent ORR catalytic activity without CO poisoning, while the CO poisoning is the major drawback in conventional Pt-based catalysts. Furthermore, the layered SiC sheets in alkaline media has better catalytic activity than Pt(111) surface and have potential as a metal-free catalyst for ORR in fuel cells.

As one of the most promising power sources, fuel cells have received considerable attention due to their high efficiency and low environmental impact. The sluggish kinetics of the oxygen reduction reaction (ORR) at the cathode is one of the key factors limiting the performance of fuel cells, and efficient ORR electrocatalysts are essential for practical applications of the fuel cells. Pt has conventionally been employed as the cathode catalyst due to its high activity for the ORR^1^,^2^. The high cost, limited supply, and poor durability of Pt catalyst however hinders the large-scale commercialization of fuel cells. Hence, during the last few decades, numerous studies have been devoted to find alternative electrocatalysts for the cathode side of fuel cells, including Pt-based alloys^3^,^4^,^5^,^6^, Pt-based core-shell/alloy nanoparticles^7^,^8^,^9^, carbon nanotubes-supported metal particles^10^,^11^, graphitic carbon nitride/carbon composite^12^,^13^ N/B/P/S doped carbon nanotubes (CNTs) and graphenes^14^,^15^,^16^.

Carbon-based catalysts are expected to be the most promising alternatives to Pt catalysts because C is more abundant and durable, as well as less expensive than Pt. The introduction of N (or B, P, S) atoms into *sp*^2^-hybridized carbon frameworks of graphenes or CNTs is generally effective in modifying their electrical properties and chemical activities^17^,^18^. Recent studies have confirmed that N, B, P and S doped carbon materials are hopeful candidates to replace Pt-based catalysts for fuel cells due to their high catalytic activity, long-term stability and excellent CO tolerance^14^,^15^,^19^,^20^,^21^,^22^,^23^. Theoretical studies have confirmed that the improved electrocatalytic activity of these materials can be attributed to the changes of electronic structure by doping N, B, P and S into carbon materials^24^,^25^. However, the dopant concentration in present carbon-based catalysts is low (N ~ 4–6 at%, B ~ 0–2.24 at%, and 1–2 at% of S)^19^,^20^,^21^, which limits the improvement of the catalytic activity of carbon-based catalysts.

SiC has a unique combination of high saturated carrier mobility, high critical electrical field, and high thermal conductivity. SiC with the atom ratio of 1:1 can provide more active reaction sites than present doped carbon materials to serve as a feasible metal-free ORR electrocatalyst. Herein, we predicted that layered SiC sheets exhibit excellent ORR catalytic activity and high CO tolerance, and may be a very likely candidate for the next generation of low-cost ORR electrocatalysts.

## Results

Firstly, the relative stability between layered and cubic SiC sheets, which is a function of layer number *N*, is determined by calculating the energy difference Δ*E* = *E*_layered_ − *E*_cubic_, where *E*_layered_ and *E*_cubic_ are the total energies of layered and cubic SiC sheets, respectively. The layered SiC sheets are energetically favorable in comparison to cubic SiC sheets when *N* < 4, while cubic SiC sheets are advantaged as *N* ≥ 4, as shown in Fig. S1 and Table S1 in supporting information. Similar to graphite, layered SiC sheets usually show weak layer-layer interactions. Even further considering the van der Waals bonding, layered SiC sheets are still energetically more favorable than cubic SiC sheets when *N* < 4. This is consistent with the recent experimental results that the thickness of synthesized 2D SiC nanosheets is between 0.5 and 1.5 nm^26^.

**Figure 1:**
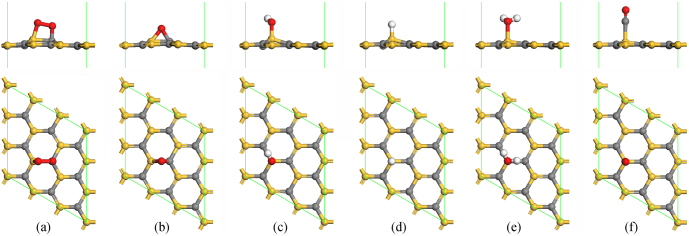
Optimized adsorption structures for ORR intermediates on a single-layer SiC: (a) O_2_, (b) O, (c) OH, (d) H, (e) H_2_O and (f) CO. Gray, gold, white and red colors denote C, Si, H and O atoms.

We then turn to consider the adsorption and reduction of O_2_ on a single-layer SiC. The adsorption energies of adsorbates on layered SiC sheets are determined by *E*_ad_ = *E*_ads_ + *E*_SiC_ − *E*_ads/SiC_, where *E*_ads_, *E*_SiC_ and *E*_ads/SiC_ are the total energies of the isolated adsorbate molecule, the layered SiC sheets and the adsorption systems, respectively. Positive adsorption energies correspond to exothermic adsorption processes, while negative adsorption energies do endothermic ones. Several possible high-symmetry adsorption sites are considered, including the top, bridge and hollow sites. The most stable adsorption structures and energies of O_2_ and ORR intermediates are summarized in Fig. 1 and Table 1. The meta-stable adsorption structures and energies are displayed in Fig. S2 of supporting information. It is found that the most energetically favorable site for O_2_ adsorption on the single-layer SiC is characterized by O_2_ nearly parallel to the SiC bond with adsorption energy of 0.56 eV. The O-O bond is elongated by 0.29 Å due to the electronic charge transfer from SiC to the 2π* orbital of O_2_, indicating that O_2_ can be dissociated easily on SiC. The charge transfer from the single-layer SiC to O_2_ molecule is quantified as 0.34 *e* according to Hirshfeld population charge distribution. Furthermore, the maximum coverage of O_2_ on SiC is also explicitly identified. As shown in Table S2 and Fig. S3 of supporting information, only two O_2_ molecules can be adsorbed on the single-layer SiC with 2 × 2 supercell, corresponding to a coverage 0.5 ML (monolayer) (1 ML is defined as one O atom per surface atom). The adsorption energy of the first O_2_ molecules on single-layer SiC is 0.48 eV. The second O_2_ molecule has two distinct adsorption structures with adsorption energies of 0.61 and 0.13 eV, respectively. It is found that the energetically more favorable configuration of second O_2_ forms two chemical bonds with Si atoms. This special adsorption structure results in the stronger adsorption of the second O_2_ molecule than the first one. This is because the electronegativity of Si (1.90) is smaller than that of C (2.55) while the Si-O bond is stronger than the C-O bond. Similar results are also found in 4 × 4 supercell, where the coverage also reaches 0.5 ML. Furthermore, O_2_ adsorbed as ordered structures in a 4 × 4 supercell is considered, as shown in Fig. S3 of supporting information, the corresponding coverage can reach 0.75 ML. However, the coverage of O_2_ on Pt(111) surface can reach 1 ML. All the O_2_ molecules adsorb on the bridge sites. This is consistent with previous theoretical work about O_2_ adsorption on Pt(111) surface^27^,^28^. Although the coverage of O_2_ on layered SiC is smaller than that on Pt(111) surface, the remaining adsorption sites on single-layer SiC is favorable for adsorption of other reactants, which may be beneficial for ORR.

The most stable adsorption site for O atom is the bridge site, which gives rise to a highly stable epoxide-like structure with adsorption energy of 4.18 eV. In addition, H and OH prefer to adsorbing at atop site of Si atom other than that of C atom with the adsorption energies of 1.32 and 2.80 eV, respectively, which differs from the case in N-doped carbon materials^29^. Because H and OH tend to adsorb at positive charged adsorption sites. Note that H_2_O is stable on atop site of Si atom of the single-layer SiC. In contrast, H_2_O dissociates to H and OH spontaneously on cubic SiC sheets, which may prevent ORR on cubic SiC sheets. The reason is that Si and C atoms are *sp*^3^ hybridized in cubic SiC sheets, while they are *sp*^2^ in layered ones. The dangling bonds of Si or C atoms at the surface of cubic SiC sheets are very active. When H is introduced to O_2_, no matter whether the presence of H_2_O molecules around, O-O bond dissociates to form O and OH spontaneously, as shown in Fig. S4 of supporting information. OOH cannot exist as a stable intermediate in the ORR processes. And then, H_2_O_2_ cannot be produced based on OOH. Thus, OOH and H_2_O_2_ pathways of ORR on the single-layer SiC are neglected here^30^,^31^.

For CO adsorption, adsorption energy value changes from −0.08 eV to 0.07 eV after considering the effect of van der Waals bonding. Since the small adsorption energy value of CO adsorption, layered SiC sheets show excellent CO tolerance. In contrast, the adsorption energy of CO on Pt(111) surface (1.86 eV) is twice as large as that of O_2_ (0.84 eV), as shown in Table S3 of supporting information. Therefore, CO blocks the active sites and hinders the ORR on Pt(111) surface^32^,^33^. From partial density of states (PDOS) (Fig. S5 of supporting information), it is found that the hybridization between CO molecule and Si atom is very small, suggesting that the interaction between CO and SiC is little. By contrast, the interaction between CO and Pt(111) surface is very strong. All the main orbitals of CO hybridize with Pt states. The renowned mechanism of CO-metal interaction, namely, via donation from CO-5σ to metals and back-donation from metals to CO-2π* orbital, is applicable for CO adsorbed on Pt(111)^34^,^35^. This is confirmed by the fact of electron depletion at CO-5σ and partially occupied CO-2π*, which is empty above the Fermi level in gas CO. Consequently, CO will poison the Pt(111) surface.

It is known that ORR mechanisms at cathodes in acidic and alkaline solutions are different. In an acidic solution, the electrode reaction can be written as O_2_ + 4H^+^ + 4e^−^ → 2O + 4H^+^ + 4e^−^ → O + OH + 3H^+^ + 3e^−^ → O + H_2_O + 2H^+^ + 2e^−^ → OH + H_2_O + H^+^ + e^−^ → 2H_2_O, while in an alkaline solution that can be expressed as O_2_ + 2H_2_O + 4e^−^ → O + 2OH^−^ + H_2_O + 2e^−^ → 4OH^−^. Both reaction pathways are considered here. In general, ORR can proceed in Langmuir-Hinshelwood (LH) or Eley-Rideal (ER) mechanisms. LH mechanism involves all the reacting intermediates on the surface, whereas ER mechanism involves species from the electrolyte reacting with a surface intermediate. Both mechanisms are also taken into account one by one.

**Figure 2:**
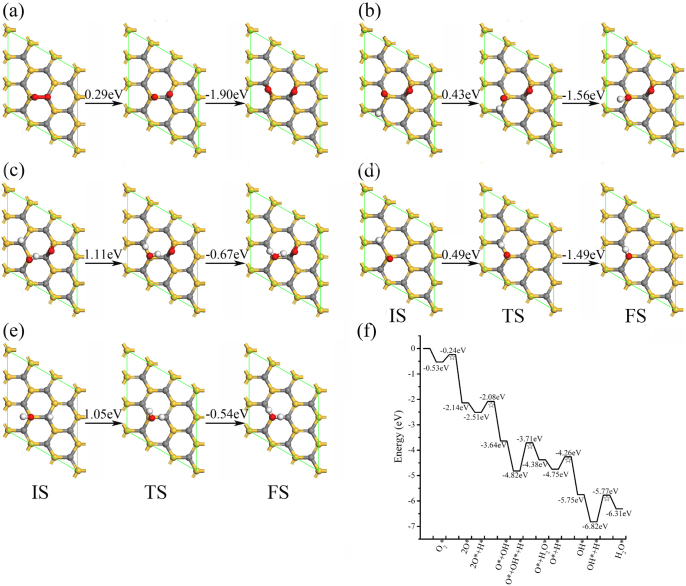
Minimum energy pathways for ORR elemental steps in acidic media on a single-layer SiC: (a) O_2_ → 2O, (b) 2O + H → O + OH, (c) O + OH + H → O + H_2_O, (d) O + H → OH, (e) OH + H → H_2_O, and (f) schematic energy profile. IS, TS and FS are initial, transition and final states, respectively. Gray, gold, white and red colors denote C, Si, H and O atoms.

The ORR following the LH mechanism is discussed firstly. The ORR mechanism on the single-layer SiC in LH mechanism under acidic media can be divided into five elemental steps: O_2_ dissociation, two O atom hydrogenation steps and two OH hydrogenation steps, as shown in Fig. 2. Some possible ORR pathways with higher barrier energies are shown in Fig. S6 of supporting information. The activation energies for the five elemental steps are 0.29 eV for O_2_ dissociation, 0.43 and 0.49 eV for first and second OH formation, 1.11 and 1.05 eV for first and second H_2_O formation, respectively. The corresponding reaction energies are −1.61, −1.13, −1.00, 0.44 and 0.51 eV. H_2_O formation is the rate-determining step (RDS) in the O_2_ dissociation mechanism of ORR in the above process. The Si-H bond broken from the initial state to the transition state contributes to the most part of the activation energy of H_2_O formation. As the number of H atoms introduced to the O atom increases, the activation energy increases from 0.49 eV to 1.05 eV and the reaction energy increases from −1 eV to 0.51 eV, corresponding to the Brønsted-Evans-Polanyi relation between activation energy and reaction energy in the heterogeneous catalysis^36^,^37^,^38^. Activation energy is a almost linear function of reaction energy, and reactions belong to the same class even follow the same relation^36^,^37^,^38^. The decrease of the activity of O atom is due to the increase of the number of introduced H atoms.

**Figure 3:**
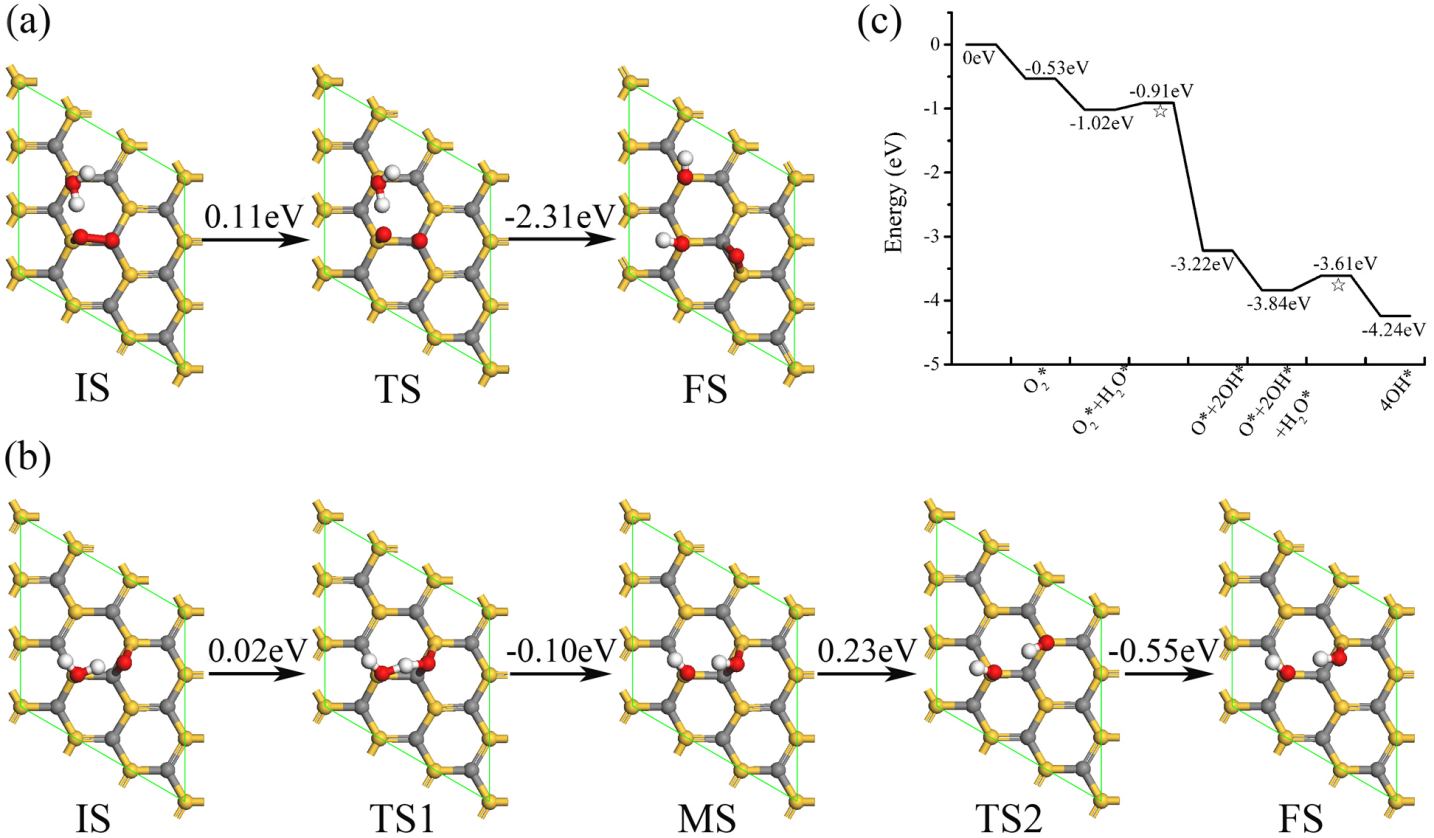
Minimum energy pathways for ORR elemental steps in alkaline media on a single-layer SiC: (a) O_2_ + H_2_O → O + 2OH, (b) O + H_2_O → 2OH, and (c) schematic energy profile. IS, TS, MS and FS are initial, transition, metastable and final states, respectively. ⋆ denotes the TS in each step. Gray, gold, white and red colors denote C, Si, H and O atoms.

ORR on the single-layer SiC in LH mechanism under an alkaline environment can be divided into the two elemental steps: O_2_ + H_2_O → O + 2OH and O + H_2_O → 2OH, as shown in Fig. 3. Differing from that in acidic environment, both steps are easy to cross with small activation energies (0.11 and 0.23 eV) and exothermic reaction energies (−2.20 and −0.40 eV). These can be deduced from the special geometrical parameters along reaction paths. O and H atoms only need a little movement from the initial state to the transition state, which costs little energy. Furthermore, the hydrogen bond between O atom and H atom in H_2_O molecule also reduce the energy barrier for H atom transfer. The activation energies for isolated H_2_O and O_2_ dissociations are 0.56 and 0.29 eV, both of them are surmountable at room temperature. This is consistent with the small activation energies for ORR elemental steps in alkaline media. Thus, we predict that ORR in LH mechanism on the single-layer SiC in the alkaline media is more favorable than that in the acidic media.

As *N* increases from 1 to 3, adsorption energies for ORR intermediates, activation and reaction energies for ORR elemental steps in LH mechanism on layered SiC sheets are almost unchanged as shown in Tables 1 and 2. Thus, layered SiC sheets with different *N* values should show similar ORR catalytic activities.

For a comparison purpose, ORR elemental steps in LH mechanism on Pt(111) surface are also calculated, as depicted in Fig. S7 and Tables S4 of supporting information. The five barriers in O_2_ dissociation mechanism are calculated to be 0.85 eV for O_2_ dissociation, 1.00 and 1.22 eV for two OH formation steps, and 0.38 eV for H_2_O formation. For the OOH association mechanism, it is found that the barriers are 0.61 eV for OOH formation, 0.03 eV for OOH dissociation, 1.22 eV for OH formation, and 0.38 eV for H_2_O formation. OH formation is RDS for both the O_2_ dissociation and OOH association mechanisms. This is consistent with the recent literature^30^. The RDS of ORR on Pt(111) surface in acidic media is the O atom hydrogenation to OH with activation energy of 1.22 eV, which is similar to that of ORR on layered SiC sheets, suggesting that layered SiC sheets and Pt(111) surface may exhibit similar activities for ORR in LH mechanism under an acidic environment. In the alkaline media, however, the activation energies for the two elemental ORR steps in LH mechanism on Pt(111) surface are 0.43 and 0.55 eV, respectively, both of them are larger than that on layered SiC sheets since the bond length of Pt-Pt (2.77 Å) is longer than that of Si-C (1.79 Å). The energy cost for the longer distance diffusion of H atom on Pt(111) surface is larger than that on layered SiC sheets. In addition, the elemental step of O + H_2_O → 2OH, which is the RDS of ORR in LH mechanism on Pt(111) surface in alkaline media, is endothermic by 0.51 eV, while that on layered SiC sheets is exothermic with reaction energy of nearly −0.40 eV, suggesting that ORR in LH mechanism on layered SiC sheets is more advantaged than that on Pt(111) surface in alkaline environment.

**Figure 4:**
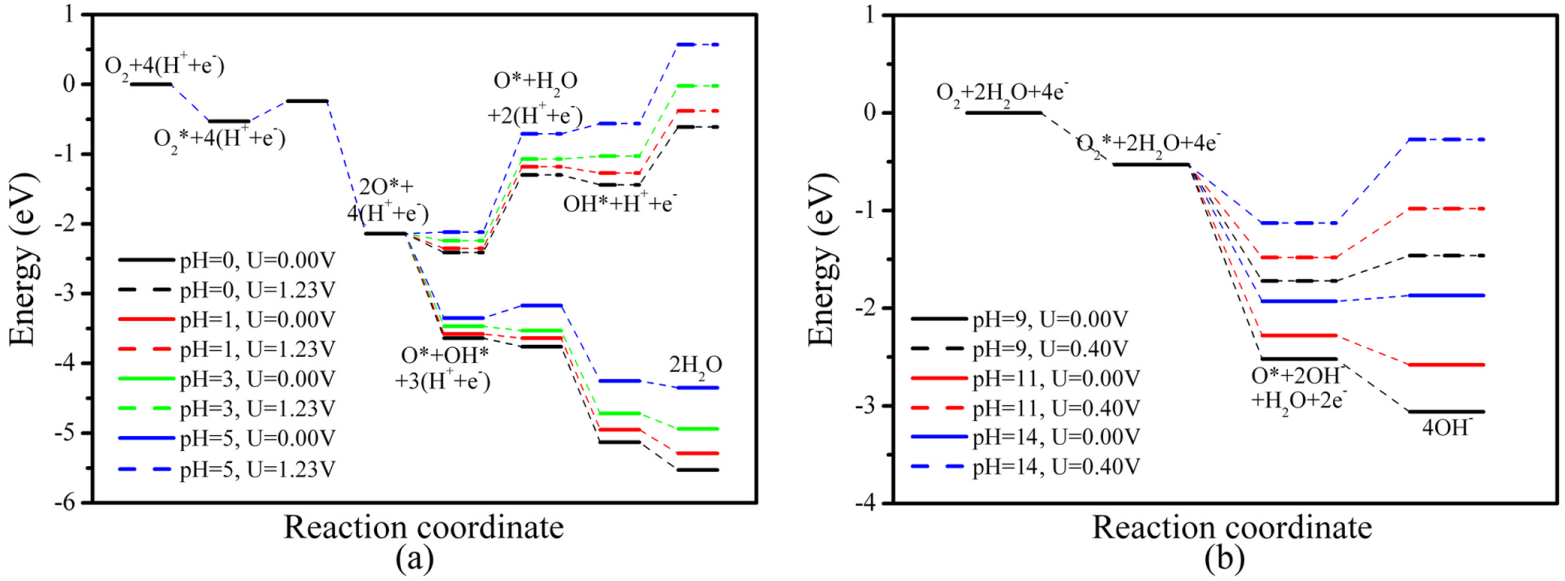
Schematic energy profile (relative to molecular O_2_ + 2H_2_ in acidic media and molecule O_2_ + 2H_2_O in alkaline media) for the ORR pathway on a single-layer SiC: (a) in acidic media, (b) in alkaline media.

Although energy diagram of ORR in ER mechanism is quite different from that in LH mechanism, both of which show similar results somehow. As shown in Fig. 4(a), ORR in ER mechanism exhibits exothermic reactivity at electrode potential *U* = 0 V under an acidic medium. As the pH value increases, the energy of each net coupled proton and electron transfer (CPET) step is shifted due to the effect of concentration on the free energy of H^+^. At pH = 1, the reaction energies of five elemental steps calculated are −1.61 eV for O_2_ dissociation, −1.44 and −1.32 eV for first and second OH formation, −0.06 and −0.34 eV for first and second H_2_O formation, respectively. When pH ≥ 4, the reaction energy of first H_2_O formation changes from exothermic to endothermic, while other steps remain exothermic. As *U* increases from 0 V to the ideal electrode potential of 1.23 V, energy levels are shifted for each net CPET step^31^,^39^. The reaction energies of the reactions in the LH mechanism and O_2_ dissociation in the ER mechanism remain unchanged. This is because no CPET step involves in these steps and the electrode potential only affects the energy levels of the electrons in the CPET steps^31^,^39^. At *U* = 1.23 V, OH formation in ER mechanism is still exothermic, while two H_2_O formation steps change to 1.17 and 0.89 eV endothermically at pH = 1, respectively. Consistent with ORR in LH mechanism, H_2_O formation is also the RDS of ORR on single SiC layer in ER mechanism at *U* = 1.23 V. The high concentration of H_2_O in electrolyte can improve the rate constant for the backward reaction and may have a negative effect on the entire process. Just as any coin has two sides, the issue being discussed here is no exception. H_2_O solution also has some positive effects on the ORR. As shown in Table S5 of supporting information, the adsorption of O_2_ is enhanced in H_2_O solution, resulting in the increase of reactant concentration on catalyst surface and forward reaction rate.

A substantially different picture is obtained for ORR in ER mechanism under alkaline environment. Similar with that under acidic media, pH imports similar effect and shifts up the energy level of every CPET step. As shown in Fig. 4(b), at *U* = 0 V, adsorbed O_2_ molecule reaction with H_2_O is exothermic with reaction energy of −1.40 eV, while O atom reaction with H_2_O is endothermic with reaction energy of 0.06 eV at pH = 14. When an ideal electrode potential of 0.40 V is applied, the energy levels of every CPET step are shifted up by 0.80 eV for every double CPET step. The reaction energies of O_2_ molecule reaction with H_2_O and O atom reaction with H_2_O vary to −0.60 and 0.86 eV at pH = 14. O_2_ molecule reaction with H_2_O is the RDS of ORR in ER mechanism under alkaline environment and the energy barrier increases as electrode potential increases.

ORR in ER mechanism on Pt(111) surface is also calculated. As shown in Fig. S8(a) of supporting information, all elemental steps involved in ORR via ER mechanism on Pt(111) are exothermic at pH = 1 and *U* = 0 V, while OH, H_2_O and OOH formations are 0.69, 0.53 and 0.63 eV endothermic at *U* = 1.23 V, which are smaller than the barrier of the RDS step (H_2_O formation) on single-layer SiC. These results are consistent with that obtained by Nørskov et al, where the reaction energy of OH and H_2_O formations change form exothermic to endothermic at *U* = 1.23 V and the OOH associative mechanism has the lowest barrier and dominates at low oxygen coverges^40^,^41^. Fig. S8(b) of supporting information also shows the energy diagram of ORR in ER mechanism on Pt(111) under alkaline environment. At *U* = 0 V and pH = 1, the reaction energies of O_2_ molecule reaction with H_2_O and O atom reaction with H_2_O on Pt(111) surface are −1.42 and 0.38 eV, respectively. At *U* = 0.40 V and pH = 1, the reaction energies of these two elemental steps vary to −0.62 and 1.18 eV, respectively. O_2_ molecule reaction with O atom is the RDS, and the barrier are larger than that on single SiC layer, suggesting that layered SiC sheets exhibit better catalytic activity than Pt(111) surface in ER mechanism under alkaline environment.

**Figure 5:**
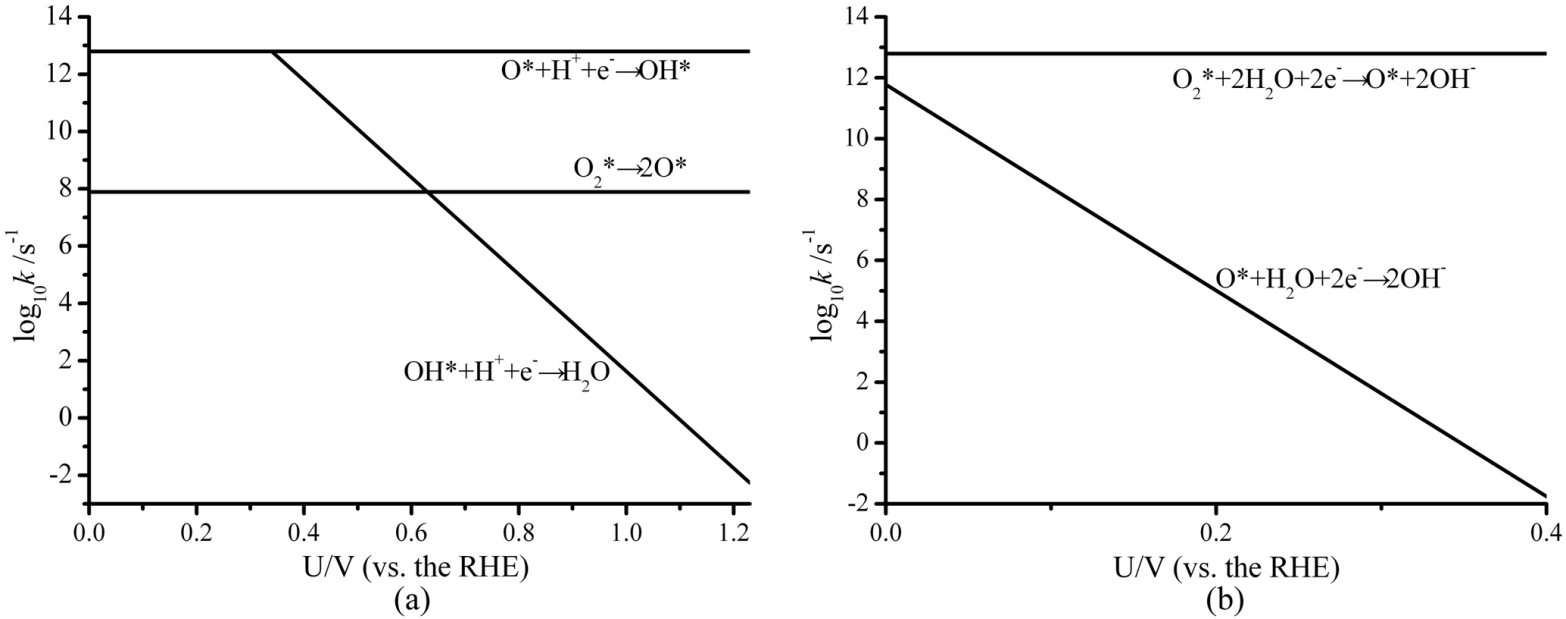
Potential-dependent rate constants for ORR on a single-layer SiC: (a) in acidic media with pH = 1, (b) in alkaline media with pH = 14.

Based on Eyring's canonical transition state theory, our calculations can be incorporated into a reduced kinetic model that should report qualitative features of the reaction mechanisms at different applied potentials^39^,^42^,^43^. As displayed in Fig. 5(a), the RDS of ORR in ER mechanism is O_2_ dissociation at low potential region, while it changes to H_2_O formation when *U* > 0.63 V at pH = 1. As shown in Fig. 5(b), O_2_ reaction with H_2_O is the RDS of ORR in ER mechanism under alkaline environment and the reaction rate decreases as electrode potential increases. Comparing Fig. 5 with Fig. S9 of supporting information, we can predict that the catalytic activity of layered SiC sheets is better than that of Pt(111) surface in alkaline media. Furthermore, due to the higher doped ratio, SiC can provide more ORR active sites than present doped carbon materials and may act as a feasible low-cost metal-free ORR electrocatalyst.

## Discussion

Since O_2_ adsorption and dissociation are the key points for ORR on layered SiC sheets, the origin of the interaction between O_2_ and the single-layer SiC is investigated by considering their electronic structure changes. Fig. S9 of supporting information illustrates the spin-polarized partial density of states (PDOS) of O-O bond on the single-layer SiC and Pt(111) surface. Generally, the 5σ, 1π and 2π* orbitals of O_2_ dominate the adsorption and all of them are broadened due to the interaction with the single-layer SiC and Pt(111) surface. The antibonding 4σ* state is not involved in the adsorption because its position is far below the Fermi level. The antibonding 2π* orbital of gas O_2_ is partially filled. After adsorption on the single-layer SiC and Pt(111) surface, the initial empty spin-down component of 2π* becomes partially occupied, inducing proportional elongation of the O-O bond length and weakening of O-O bond strength^44^,^45^. The electron occupying the antibonding 2π* orbital of adsorbed O_2_ on the single-layer SiC is 1.55 *e* compared with 1.19 *e* on Pt(111) surface and 1.07 *e* in gas phase according to integrated DOS. Thus, the activation energy of O_2_ on the single-layer SiC are smaller than that on Pt(111) surface.

In summary, our DFT calculations suggest that novel metal-free layered SiC sheets with *N* = 1 ~ 3 can exist stably and possess potential ORR catalytic activity due to two advantages of layered SiC sheets compared to Pt(111) surface: (i) free from CO poisoning, and (ii) lower activation energies for the RDS of ORR on layered SiC sheets in alkaline media than that on Pt(111) surface. In addition, the corresponding electronic structures are analyzed. The results show that the layered SiC sheets are candidates for practical applications in fuel cells.

## Methods

All calculations are performed within density functional theory (DFT) framework as implemented in DMol^3^ code^46^,^47^. The All Electron Relativistic core treat method is implemented for relativistic effects, which explicitly includes all electrons and introduces some relativistic effects into the core. The generalized gradient approximation (GGA) with Perdew−Burke−Ernzerhof (PBE) functional is employed to describe exchange and correlation effects^48^. PBE is the most common density functional in materials and surface science. However, it cannot accurate describe the van der Waals forces^49^,^50^,^51^,^52^. This is mainly because the GGA-PBE fails to describe non-local dispersion forces, which are expected to be relevant in layered inorganic compounds and weak adsorption systems. The lack of inclusion of long range correlation in the local density functional (LDF) calculations prevents the accurate calculation of the van der Waals bonds^46^. Therefore, a Grimme approach is adopted for dispersion corrections. Dmol^3^ uses a double set of numerically tabulated basis functions^46^. A more precise term would be “double numerical basis” to be contrasted with double zeta basis, where the radial functions are defined as Slater zeta functions. The basis set can be significantly improved by adding higher angular momentum valence polarization functions and also by core polarization functions^46^. Total energy for the double basis is quite uniformly higher than the ones for the extended basis sets. It has shown that the basis set produces errors in self-consistent eigenvalues and total energy of ≈0.00003 Ha. In this work, the double numerical atomic orbital augmented by a polarization *p*-function (DNP) is chosen as the basis set^46^. The accuracy of DNP is comparable to a Gaussian 6-31(d) basis^53^. The double basis set may be considered as a large basis especially for the larger molecules. Recent theoretical works based this basis set have shown excellent consistency with experiments^54^,^55^. We have also compared our results with that obtained based on triple numerical plus polarization (TNP), as shown in Tables S6 and S7. It can be found that the adsorption energies based on TNP are slightly larger than that based on DNP, while the activation energies and reaction energies of ORR in LH mechanism based on both basis sets are almost the same. A smearing of 0.005 Ha (1 Ha = 27.21 eV) to the orbital occupation is applied to achieve accurate electronic convergence. The spin-unrestricted method is used for all calculations. To ensure high-quality results, the real-space global orbital cutoff radius is chosen as high as 4.6 Å in the computations. The k-point density is set as 2/3 × 2/3 × 1 for per unit cell. The convergence tests for k-point density are shown in Table S8 of supporting information, which shows that the energy of single SiC layer is converging when k-point density is larger than 2/3 × 2/3 × 1. The convergence tolerance of energy is 1.0 × 10^−5^ Ha, maximum force is 0.002 Ha/Å, and maximum displacement is 0.005 Å in the geometry optimization. The transition states for ORR elemental steps are obtained by LST/QST tools in DMol^3^ code. Frequency calculations are performed to confirm the location of the transition states. A conductor-like screening model (COSMO) is used to simulate a H_2_O solvent environment throughout the whole process. All data, except for clearly stated, are obtained under this method. The COSMO is a continuum model in which the solute molecule forms a cavity within the dielectric continuum of permittivity^56^. The DMol^3^/COSMO method has been generalized to the periodic boundary cases. The deviation of this COSMO approximation from the exact solution is small. For strong dielectrics like H_2_O, it is less than 1%. The dielectric constant is set as 78.54 for H_2_O solvent. Our previously theoretical work, focused on ORR on N doped CNT based on COSMO, is consistent with experiments well^29^. In addition, some other results also show that this implicit solvation model is an effective method to describe the solvation^30^,^31^. The solvation energies of ORR intermediates on SiC sheets are shown in Table S9 of supporting information and adsorption energies in a gas phase environment are shown in Table S5 of supporting information, from which we can find that O_2_ and OH are stabilized on SiC sheets by solvation. For layered SiC sheets, all simulations are performed in a 3 × 3 supercell except for coverage examination of O_2_ adsorption, where 2 × 2 and 4 × 4 supercells are used. The minimal distance between the SiC layers and their mirror images is set as 15 Å, which is sufficiently large to avoid the interaction between them. The Si-C bond length is 1.79 Å. The Pt(111) surface is modeled by a periodic array of Pt slabs with a vacuum width in excess of 15 Å. The p(3 × 3) unit cells with three layer Pt slabs are used throughout in the ORR calculations. The bottom two Pt layers are fixed at their bulk positions and the top Pt layer is allowed to relax fully. Tests performed with four and five atomic layers, top two of which are allowed to relax, do not show significant differences in structural parameters. As shown in Table S10 of supporting information, the adsorption energy variation for adsorbed molecules is smaller than 0.09 eV.

In this work, both LH and ER mechanisms are expected to proceed. The LH reaction referrs to the reaction of adsorbed hydrogen atoms with another adsorbate, and the ER reaction does the reaction of a proton from solution interacting with an adsorbate. The complete electrochemical ORR in ER mechanism involves four CPETs to O_2_ molecule at the cathode^31^,^39^. Electron transfers are coupled with a proton transfer as well. Barriers for electrochemical proton-transfer have been calculated for the reduction of O_2_ to OOH and OH to H_2_O on Pt^57^,^58^. In both cases, the proton-transfer reaction barrier calculated is small (0.15 eV to 0.25 eV) at the low potential that elementary steps are exothermic, and diminishes with higher applied voltages^57^,^58^. Similarly, as a first approximation, we expect also that barriers for electrochemical proton transfers to adsorbed species will be small and be easily surmountable at room temperature. As a result, we only calculated reaction energy for ORR in the ER mechanism. Free energies of the ORR intermediates are calculated based on a computational hydrogen electrode (CHE) model suggested by Nøskov et al^40^,^59^. Free energy change (Δ*G*) is determined by Δ*G* = Δ*E* + Δ*ZPE* − *T*Δ*S* + Δ*G*_pH_ + Δ*G*_U_, where Δ*E* is the reaction energy, Δ*ZPE* is the zero point energy, *T* is temperature and Δ*S* is the change in the entropy. Δ*G*_pH_ and Δ*G*_U_ are the free energy contributions due to variations in H^+^ concentration and electrode potential, *U*, respectively. In this work, we consider the contributions of Δ*E*, Δ*G*_pH_ and Δ*G*_U_ to free energy and neglect the effects of other terms^60^. Effects of other terms will be included in a forthcoming publication. We assume pH = 0, 1, 3 and 5 for acidic medium and pH = 9, 11 and 14 for alkaline medium. The pH effect is very hard to be considered directly in the electrolyte. Generally, it can be treated following the method directed by Nørskov et al^40^. At a pH differing from 0, the free energy of H^+^ ions can be corrected by the concentration: *G*_pH_ = −*k*_B_*T*ln[H^+^] = pH × *k*_B_*T*ln10. This pH-dependence effect does not enter the COSMO-approach.

Naturally, ORR is a highly complicated process. Undertaking a kinetics analysis using rate constants derived from first-principles calculations would be an ideal way to determine which pathways are relevant under different conditions. Such a kinetics analysis is unfortunately difficult, since it would require rigorous double-layer effects, such as intermediate concentrations, surface coverages, or the potential drop within the interface. At here, the potential dependent rate constants *k*(*U*) are obtained based on Eyring's canonical transition state theory: 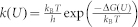, where *k*_B_ is Boltzmann constant, *T* = 298.15 K, *h* is Planck constant, and Δ*G*(*U*) is the potential-dependent barrier for that process^39^,^42^,^43^.

## Author Contributions

P.Z. conceived the initial idea of this research, performed the computer simulations and wrote the paper. B.B.X., X.L.H., Y.F.Z. and Q.J. participated in the discussion and revised the manuscript. Y.F.Z. and Q.J. guided the work.

## Supplementary Material

Supplementary Information Layered SiC Sheets: A Potential Catalyst for Oxygen Reduction Reaction

## Figures and Tables

**Table 1:** Adsorption energy (*E*_ad_) values of ORR intermediates on layered SiC sheets. All results are in unit of eV. SiC-N denotes N layered SiC sheet

	O_2_	O	OH	H	H_2_O	CO
SiC-1	0.53	4.12	2.80	1.32	0.17	−0.08
	(0.66)^a^	(4.20)^a^	(2.92)^a^	(1.38)^a^	(0.32)^a^	(0.07)^a^
SiC-2	0.46	4.09	2.93	1.41	0.18	−0.09
SiC-3/ABA	0.50	4.11	2.87	1.35	0.15	−0.07
SiC-3/ABC	0.56	4.14	2.86	1.35	0.10	−0.09

^a^The energies in parenthesis are performed under the consideration of van der Waals bonding.

**Table 2:** The activation energies (*E*_a_) and reaction energies (*E*_r_) for elemental steps involved in ORR in LH mechanism on layered SiC sheets. All results are in unit of eV. SiC-N denotes N layered SiC sheet

	SiC-1		SiC-2		SiC-3/ABA		SiC-3/ABC	
Reaction steps	*E*_a_	*E*_r_	*E*_a_	*E*_r_	*E*_a_	*E*_r_	*E*_a_	*E*_r_
O_2_ → 2O	0.29	−1.61	0.27	−1.60	0.29	−1.59	0.29	−1.59
2O + H → OH + O	0.43	−1.13	0.38	−1.21	0.40	−1.18	0.41	−1.13
O + OH + H → H_2_O + O	1.11	0.44	1.09	0.39	1.08	0.41	1.09	0.45
O + H → OH	0.49	−1.00	0.45	−1.12	0.45	−1.06	0.47	−0.99
OH + H → H_2_O	1.05	0.51	1.02	0.46	1.03	0.50	1.04	0.54
O_2_ + H_2_O → O + 2OH	0.11	−2.20	0.09	−3.25	0.09	−2.29	0.08	−2.32
O +H_2_O → 2OH	0.23	−0.40	0.20	−0.53	0.23	−0.46	0.21	−0.48
