# Supplementary material for: Layered SiC Sheets: A Potential Catalyst for Oxygen Reduction Reaction
Source: Sci Rep. 2014 Jan 22;4:3821. doi: 10.1038/srep03821 (PMC3898266; doi:10.1038/srep03821)
Supplement: Supplementary Information — Layered SiC Sheets: A Potential Catalyst for Oxygen Reduction Reaction [file srep03821-s1.pdf]

Supporting Information

Layered SiC Sheets: A Potential Catalyst for Oxygen Reduction Reaction

P. Zhang[1,2], B. B. Xiao[1], X. L. Hou[1,2], Y. F. Zhu[1],* Q. Jiang[1*]

[1]*Key Laboratory of Automobile Materials, Ministry of Education, and Department of Materials Science and Engineering, Jilin University, Changchun 130022, China,*

[2]*Institute for Advanced Materials, and School of Materials Science and Engineering, Jiangsu University, Zhenjiang 212013, China.*

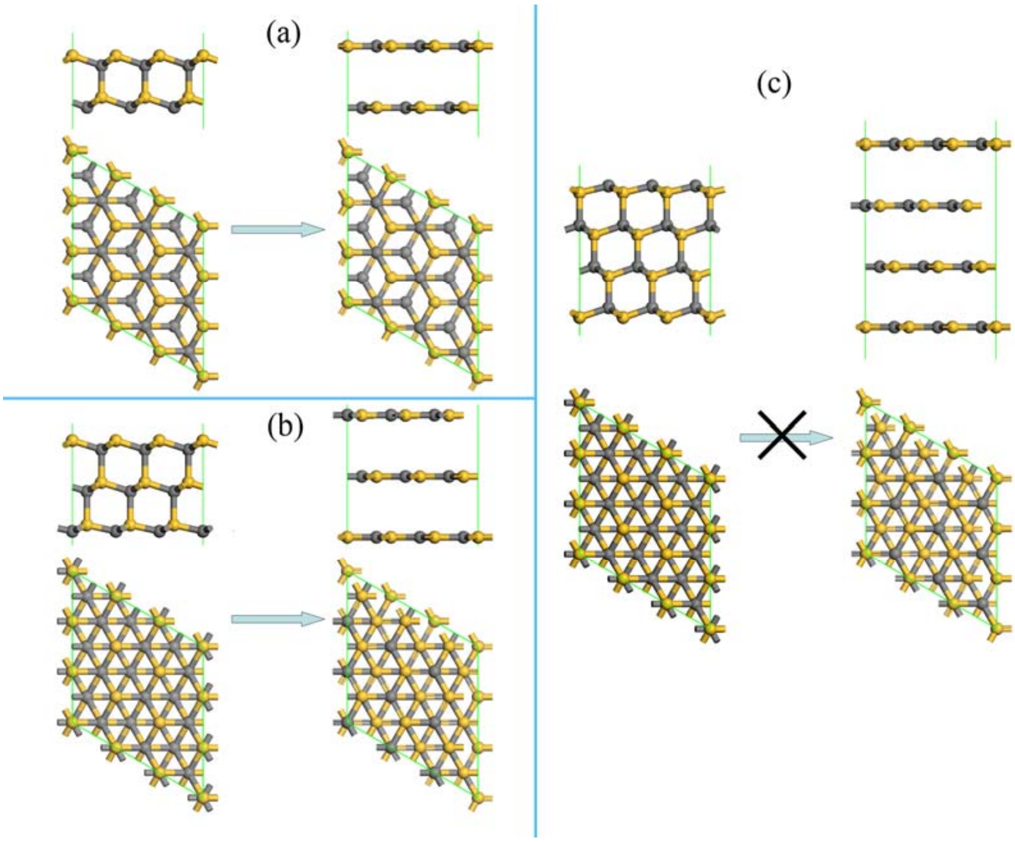

Figure S1. Schematic diagrams of layered and cubic SiC sheets: (a) two-layer SiC sheet (SiC-2), (b) three-layer SiC sheet (SiC-3) and (c) four-layer SiC sheet (SiC-4). Gray and gold colors denote C and Si atoms.

* Corresponding author. Fax: 86-431-85095371; Email: yfzhu@jlu.edu.cn, jiangq@jlu.edu.cn

Table S1. The relative stability between cubic and layered SiC sheets: two-layer SiC sheet (SiC-2), three-layer SiC sheet (SiC-3), four-layer SiC sheet (SiC-4) and five-layer SiC sheet (SiC-5). All results are in unit of eV.

		SiC-2	SiC-3	SiC-4	SiC-5
ΔE/eV	ABA	-[a]	–0.47	0.47	1.23
	ABC	-[a]	–0.48	0.47	1.23
(ΔE/eV)[b]	(ABA)[b]	(-)[b]	(–0.27)[b]	(0.61)[b]	(1.69)[b]
	(ABC)[b]	(-)[b]	(–0.26)[b]	(0.61)[b]	(1.70)[b]

[a] When $N = 2$, cubic SiC sheet transforms to layered SiC sheet spontaneously after structure optimization. [b] The energies in parenthesis are based on DFT including van der Waals bonding.

Table S2. Adsorption energy (E_{ad}) values for three O_2 molecules adsorbed on single-layer SiC and Pt(111) surface in sequence.

O_2	1st	2nd	3rd
SiC-1	0.48	0.61	0
Pt(111)	0.92	0.80	0

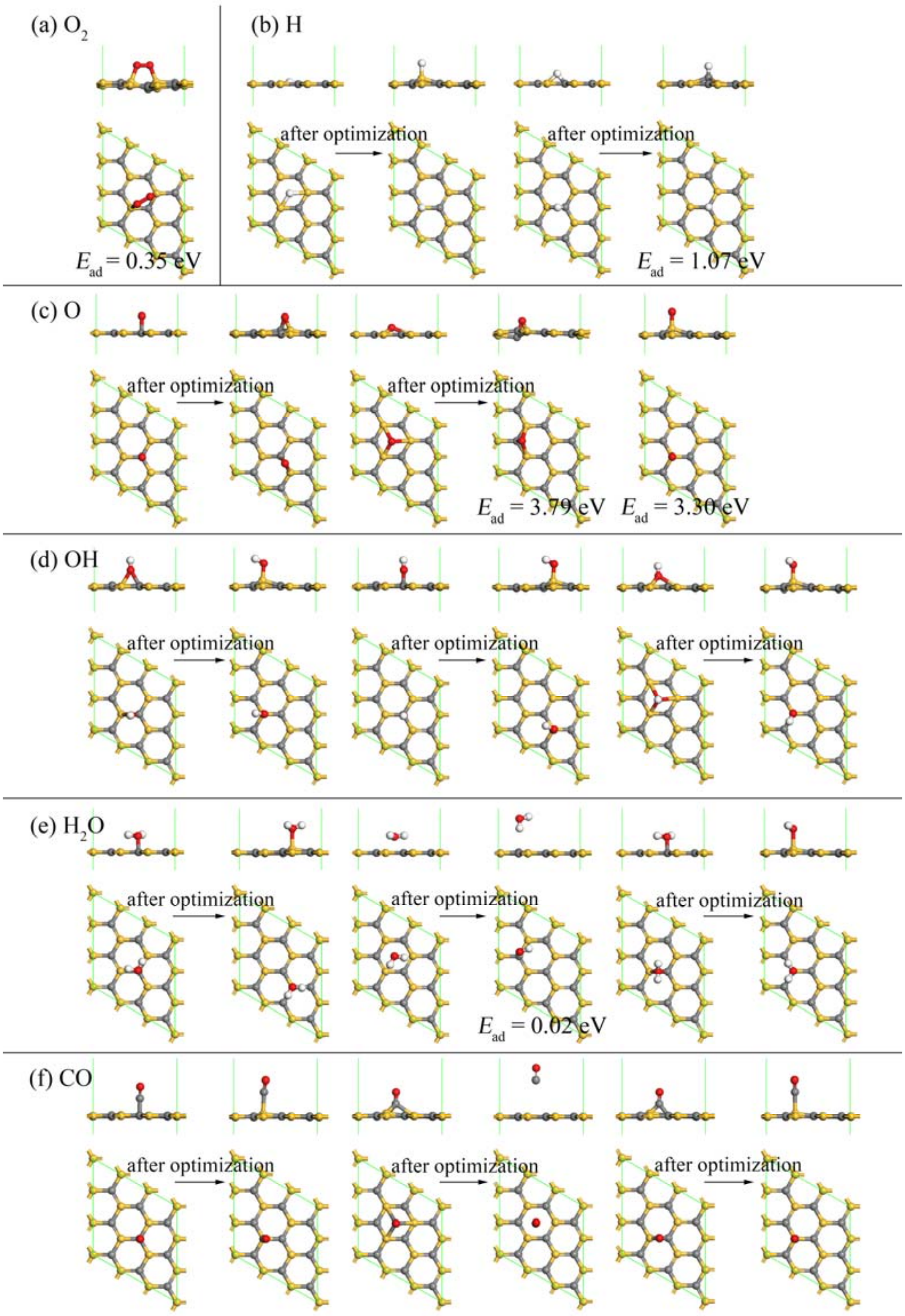

Figure S2. Structures of O_2 and ORR intermediates adsorbed on single-layer SiC before and after optimization. Optimized adsorption structures for ORR intermediates on a single-layer SiC: (a) O_2, (b) H, (c) O, (d) OH, (e) H_2O and (f) CO. Gray, gold, white and red colors denote C, Si, H and O atoms.

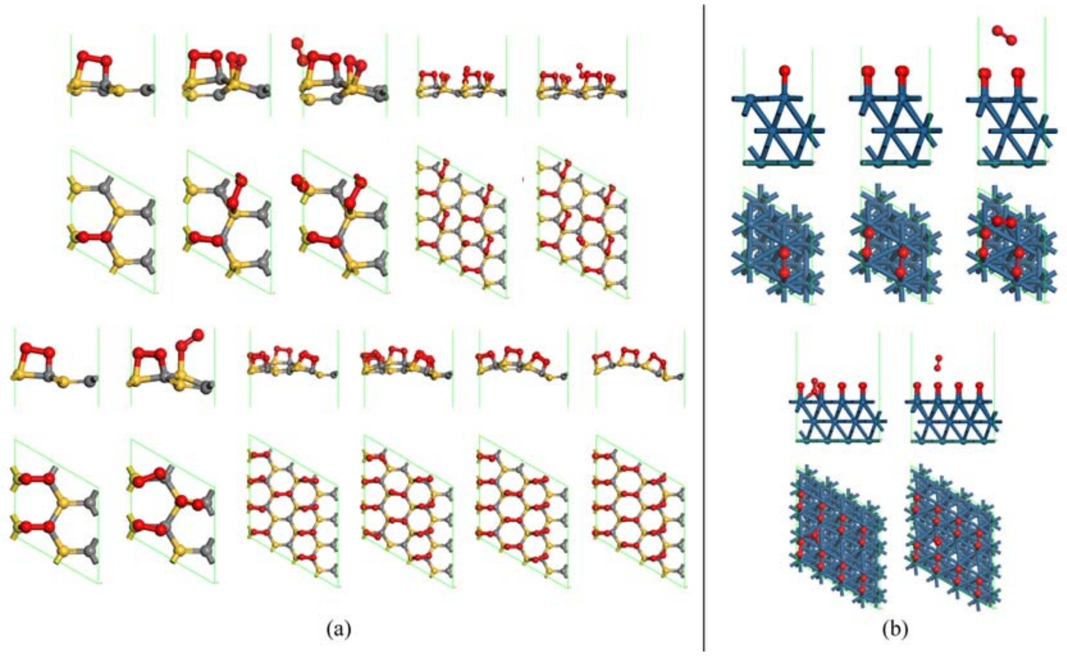

Figure S3. Optimized adsorption structures for O_2 molecules adsorbed on single-layer SiC (a) and Pt(111) surface (b) in sequence. Gray, gold, white, red and blue colors denote C, Si, H, O and Pt atoms.

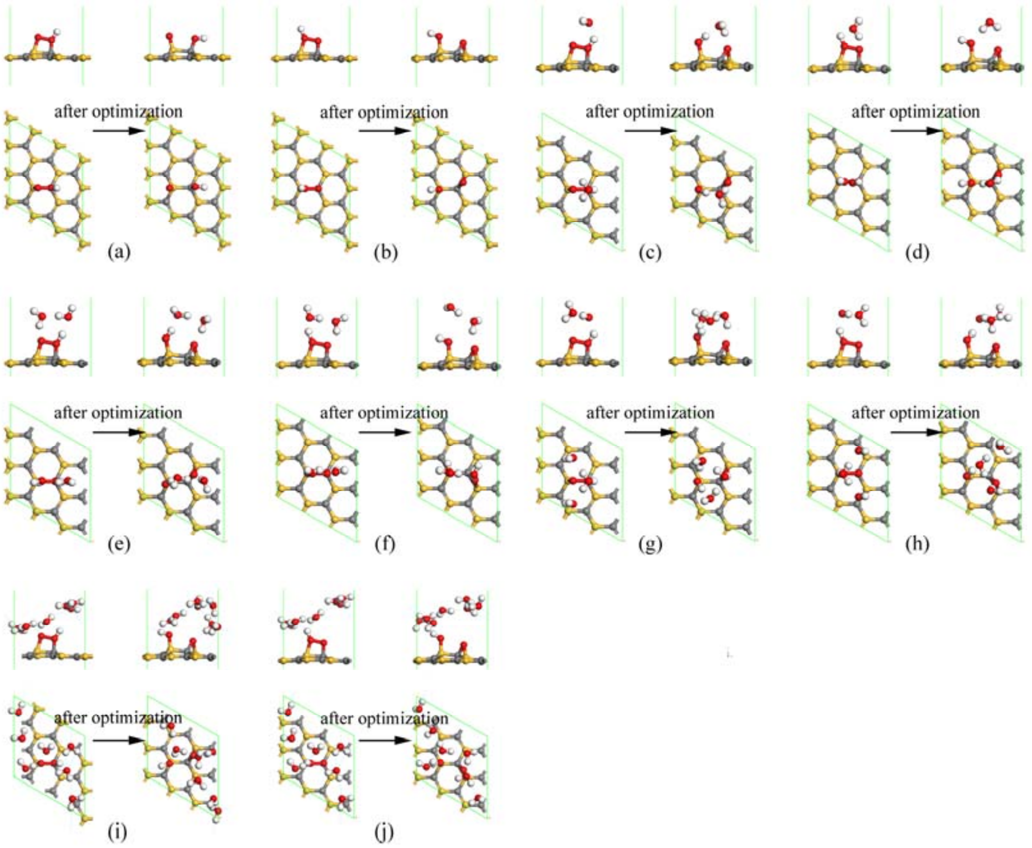

Figure S4. Structures of OOH adsorbed on single-layer SiC before and after optimization. (a) and (b) do not contain H_2O molecules, (c) and (d) have one H_2O molecule, (e) and (f) have two H_2O molecules, (g) and (h) have three H_2O molecules, (i) and (j) have seven H_2O molecules. Gray, gold, white and red colors denote C, Si, H and O atoms.

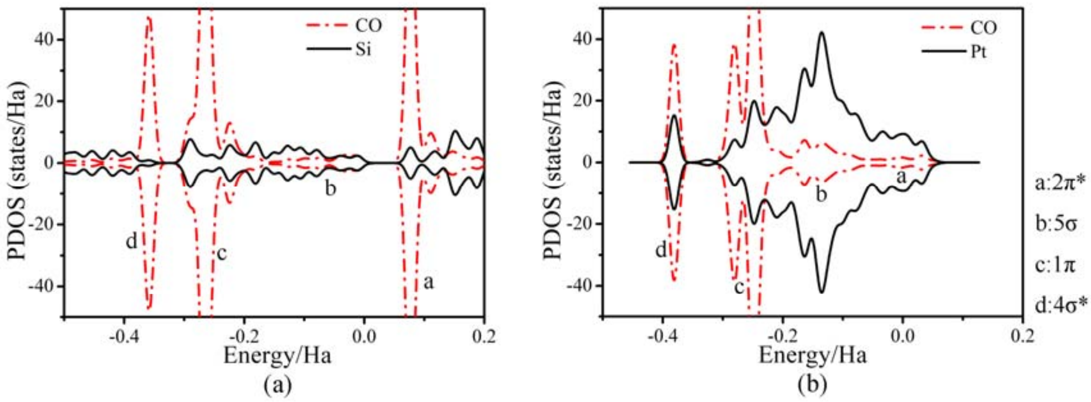

Figure S5. Partial density of states (PDOS) for CO adsorbed on single-layer SiC (a) and Pt(111) surface (b).

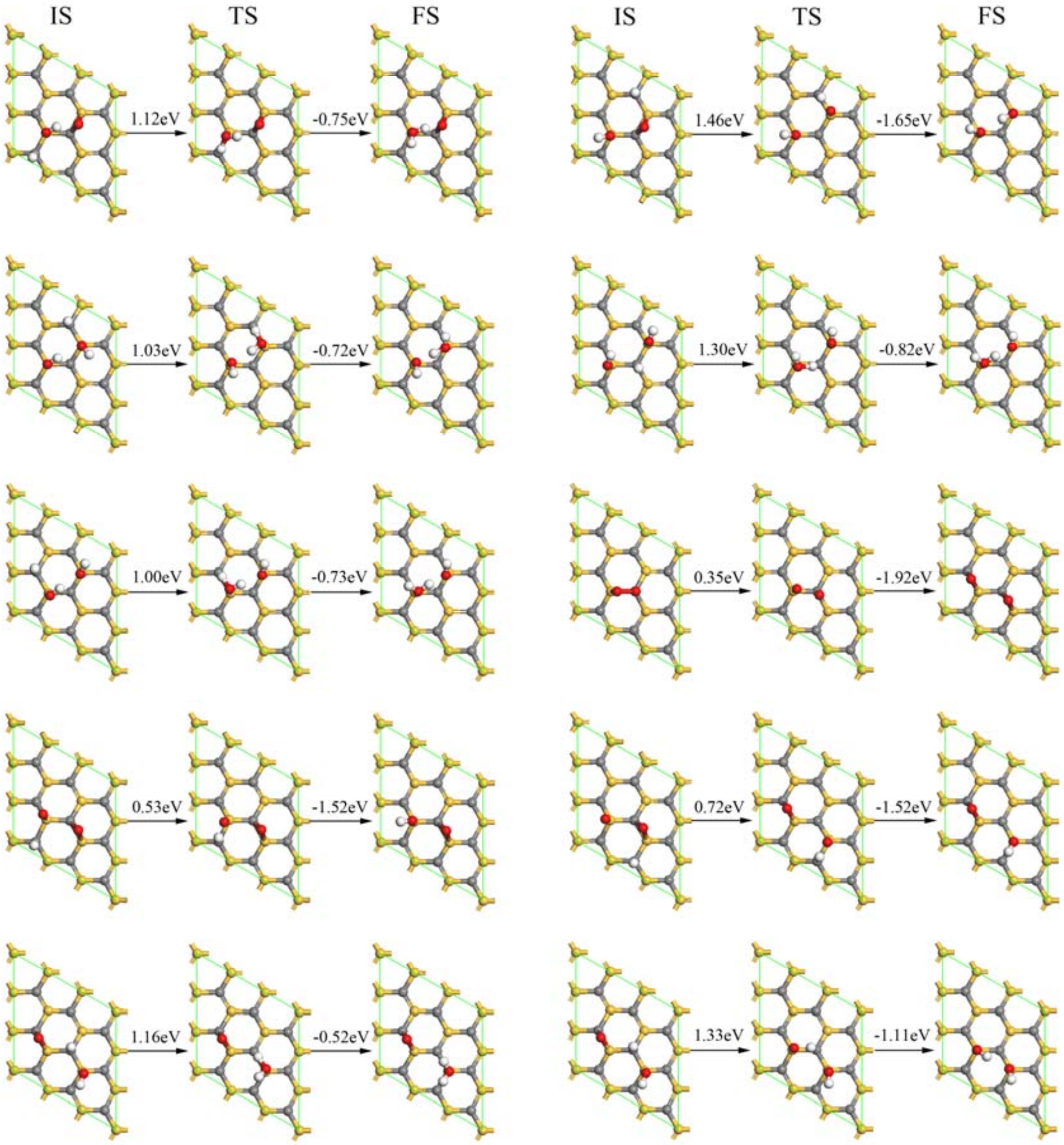

Figure S6. Initial states (IS), transition states (TS), and final states (FS) for some possible paths of ORR on single-layer SiC. Gray, gold, white and red colors denote C, Si, H and O atoms.

Table S3. Adsorption energy (E_{ad}) values of ORR intermediates on Pt(111) surface. All results are in unit of eV.

adsorbates	O_2	O	OH	OOH	H	H_2O	CO
E_{ad}	0.84/bri	4.45/fcc	2.36/top	1.28/bri	3.05/top	0.46/top	1.86/fcc

Table S4. The activation energies (E_a) and reaction energies (E_r) for elemental steps in ORR on Pt(111) surface. All results are in unit of eV.

Reaction steps	Pt(111)	
	E_a	E_r
$O_2 \rightarrow 2O$	0.85, 0.37,[a] 0.65,[b] 0.58[d]	–1.57, –1.05[b]
$2O + H \rightarrow O + OH$	1.00	–0.04
$O + OH + H \rightarrow O + H_2O$	0.38	–0.59
$O + H \rightarrow OH$	1.22, 0.91,[a] 1.25,[b] 0.96,[c] 0.73[d]	0.14, –0.20,[a] –0.65,[b] 0.06[c]
$OH + H \rightarrow H_2O$	0.38, 0.14,[a] 0.24,[b] 0.21,[c] 0.21[d]	–0.57, –0.75,[a] –1.05,[b] –0.47[c]
$O_2 + H \rightarrow OOH$	0.61, 0.28[d]	0.04
$OOH \rightarrow O + OH$	0.03, 0.00[d]	–1.22
$O_2 + H_2O \rightarrow O + 2OH$	0.43	–0.65
$O + H_2O \rightarrow 2OH$	0.55, 0.33[c]	0.51, 0.20[c]

[a] Reference 1. [b] Reference 2. [c] Reference 3. [d] Reference 4.

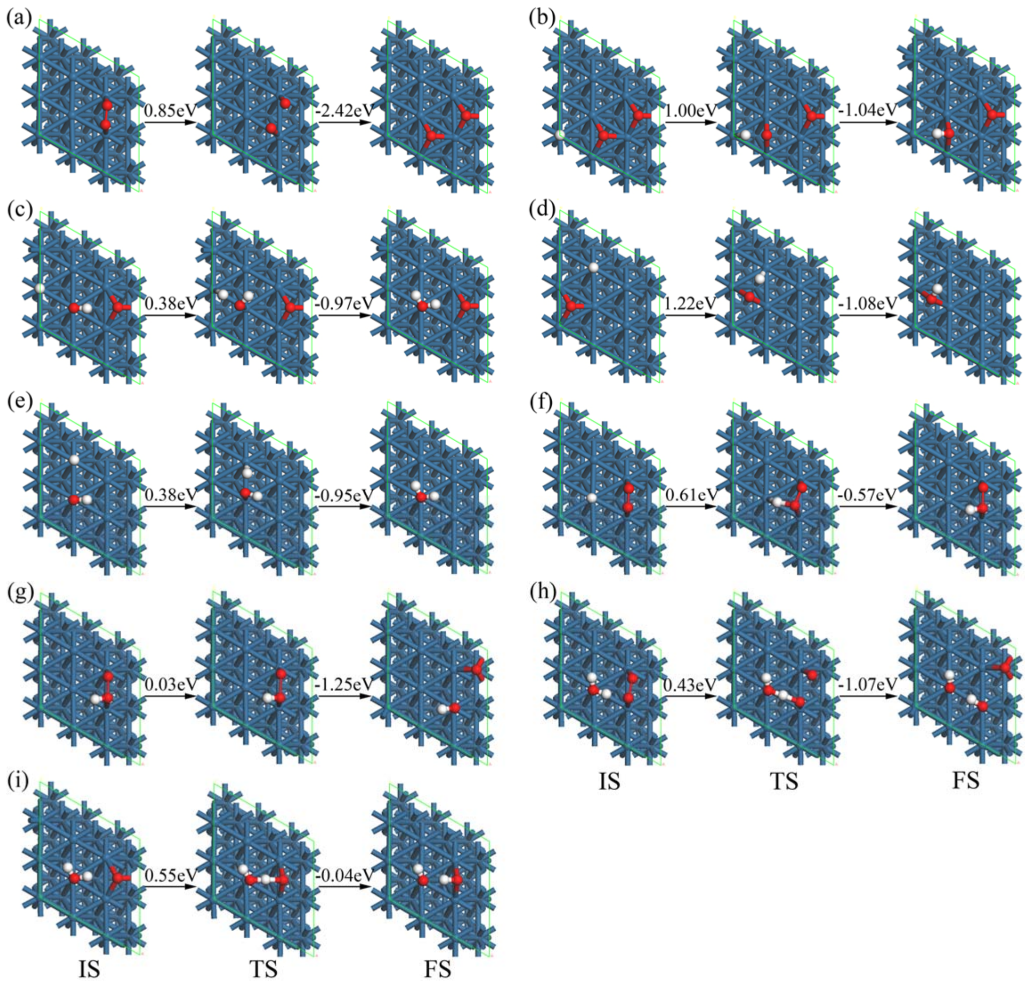

Figure S7. Minimum energy pathways for ORR elemental steps on Pt(111) surface: (a) $O_2 \rightarrow 2O$, (b) $2O + H \rightarrow O + OH$, (c) $O + OH + H \rightarrow O + H_2O$, (d) $O + H \rightarrow OH$, (e) $OH + H \rightarrow H_2O$, (f) $O_2 + H \rightarrow OOH$, (g) $OOH \rightarrow O + OH$, (h) $O_2 + H_2O \rightarrow O + 2OH$, and (i) $O + H_2O \rightarrow 2OH$. IS, TS and FS are initial, transition and final states, respectively. Blue, white and red colors denote Pt, H and O atoms.

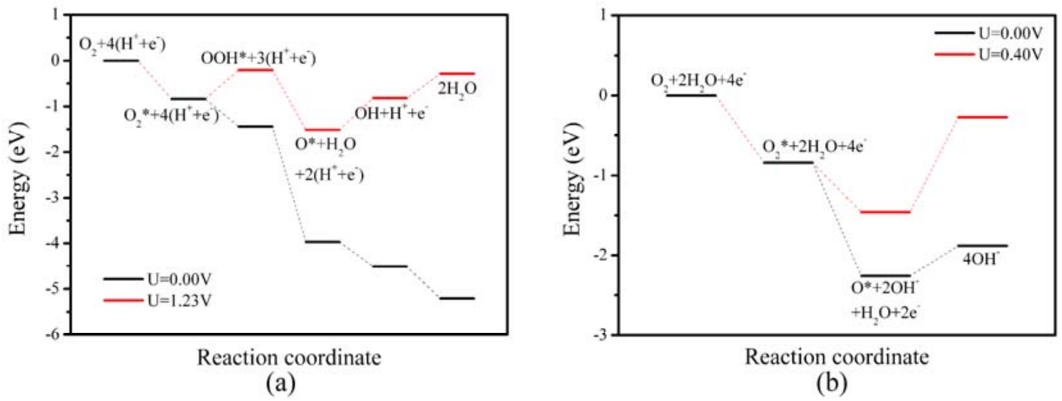

Figure S8. Schematic energy profile (relative to molecular O_2 + $2H_2$ in acidic media and molecule O_2 + $2H_2O$ in alkaline media) for the ORR pathway on Pt(111) surface: (a) in acidic media with pH = 1, (b) in alkaline media with pH = 14.

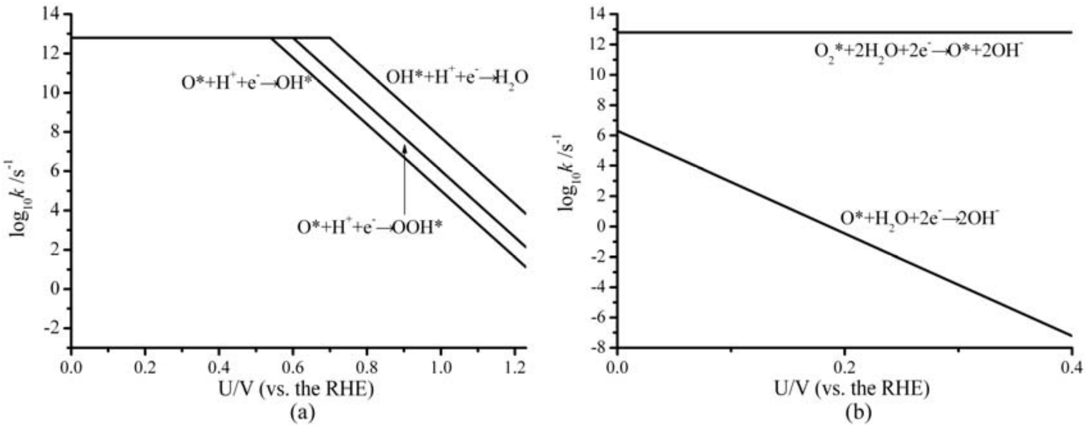

Figure S9. Potential-dependent rate constants for ORR on Pt(111) surface: (a) in acidic media with pH = 1, (b) in alkaline media with pH = 14.

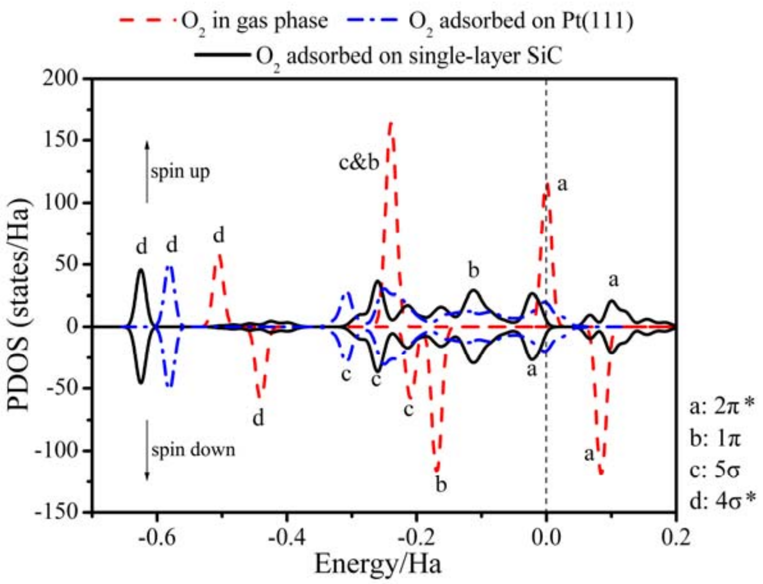

Figure S10. Partial density of states (PDOS) for O_2 adsorded on single-layer SiC and Pt(111) surface.

Table S5. Adsorption energy (E_{ad}) values of ORR intermediates on layered SiC sheets in the gas-phase environment. All results are in unit of eV. SiC-N denotes N layered SiC sheets.

	O_2	O	OH	H	H_2O	CO
SiC-1	0.36	4.03	2.82	1.31	0.18	–0.09
SiC-2	0.35	4.03	2.96	1.41	0.19	–0.10
SiC-3/ABA	0.38	4.04	2.89	1.34	0.19	–0.07
SiC-3/ABC	0.40	4.05	2.86	1.33	0.18	–0.07

Table S6. Comparison of the adsorption energies (E_{ad}) for ORR intermediates on single-layer SiC based on different basis set (DNP and TNP). All results are in unit of eV.

E_{ad}	O_2	O	OH	H	H_2O	CO
DNP	0.53	4.12	2.80	1.32	0.17	–0.08
TNP	0.78	4.27	2.89	1.32	0.18	–0.01

Table S7. Comparison of the activation energies (E_a) and reaction energies (E_r) of three ORR elemental steps in LH mechanism based on different basis set (DNP and TNP). All results are in unit of eV.

Reaction steps	DNP		TNP	
	E_a	E_r	E_a	E_r
$O_2 \rightarrow 2O$	0.29	–1.61	0.24	–1.71
$O + H \rightarrow OH$	0.49	–1.00	0.49	–0.99
$OH + H \rightarrow H_2O$	1.05	0.51	1.05	0.54

Table S8. Selected results for the convergence test of k point density. All results are in unit of Ha.

k point density	1/3×1/3×1	2/3×2/3×1	1×1×1	2×2×1
SiC-1	–2952.0874	–2952.1478	–2952.1476	–2952.1476
O_2 adsorbed on SiC-1	–3102.6782	–3102.7266	–3102.7264	–3102.7264

Table S9. Solvation energy (E_{solv}) for all intermediates on layered SiC sheets. All results are in unit of eV.

	O_2	O	OH	H	H_2O	CO
	–0.01	0	–0.05	0	–0.31	–0.02
SiC-1	SiC-1+O_2	SiC-1+O	SiC-1+OH	SiC-1+H	SiC-1+H_2O	SiC-1+CO
–0.04	–0.21	–0.13	–0.20	–0.05	–0.33	–0.07
SiC-2	SiC-2+O_2	SiC-2+O	SiC-2+OH	SiC-2+H	SiC-2+H_2O	SiC-2+CO
–0.04	–0.15	–0.10	–0.19	–0.04	–0.34	–0.07
SiC-3 (ABA)	SiC-3+O_2	SiC-3+O	SiC-3+OH	SiC-3+H	SiC-3+H_2O	SiC-3+CO
–0.04	–0.16	–0.10	–0.20	–0.04	–0.30	–0.06
SiC-3 (ABC)	SiC-3+O_2	SiC-3+O	SiC-3+OH	SiC-3+H	SiC-3+H_2O	SiC-3+CO
–0.05	–0.21	–0.14	–0.23	–0.07	–0.28	–0.06

Table S10. Selected results of the layer convergence tests for OH, CO and H adsorption on Pt(111) surface. All results are in unit of eV.

Layers	3	4	5
OH	2.36	2.29	2.31
CO	2.00	1.92	1.93
H	3.04	2.95	2.98

REFERENCES

1. Qi, L., Yu, J. & Li, J. Coverage dependence and hydroperoxyl-mediated pathway of catalytic water formation on Pt (111) surface. *J. Chem. Phys.* **125**, 054701 (2006).

2. Keith, J. A., Jerkiewicz, G. & Jacob, T. Theoretical investigations of the oxygen reduction reaction on Pt(111). *ChemPhysChem* 11, 2779-2794 (2010).

3. Michaelides, A. & Hu, P. Catalytic water formation on platinum: a first-principles study. *J. Am. Chem. Soc.* **123**, 4235-4242 (2001).

4. Sha, Y., Yu, T. H., Liu, Y., Merinov, B. V. & Goddard, W. A. Theoretical study of solvent effects on the platinum-catalyzed oxygen reduction reaction. *J. Phys. Chem. Lett.* **1**, 856-861 (2010).
